# Optimal Use of Conservation and Accessibility Filters in MicroRNA Target Prediction

**DOI:** 10.1371/journal.pone.0032208

**Published:** 2012-02-27

**Authors:** Ray M. Marín, Jiří Vaníček

**Affiliations:** Laboratory of Theoretical Physical Chemistry, Institut des Sciences et Ingénierie Chimiques, École Polytechnique Fédérale de Lausanne, Lausanne, Switzerland; French National Center for Scientific Research - Institut de Biologie Moléculaire et Cellulaire, France

## Abstract

It is generally accepted that filtering microRNA (miRNA) target predictions by conservation or by accessibility can reduce the false discovery rate. However, these two strategies are usually not exploited in a combined and flexible manner. Here, we introduce PACCMIT, a flexible method that filters miRNA binding sites by their conservation, accessibility, or both. The improvement in performance obtained with each of these three filters is demonstrated on the prediction of targets for both i) highly and ii) weakly conserved miRNAs, i.e., in two scenarios in which the miRNA-target interactions are subjected to different evolutionary pressures. We show that in the first scenario conservation is a better filter than accessibility (as both sensitivity and precision are higher among the top predictions) and that the combined filter improves performance of PACCMIT even further. In the second scenario, on the other hand, the accessibility filter performs better than both the conservation and combined filters, suggesting that the site conservation is not equally effective in rejecting false positive predictions for all miRNAs. Regarding the quality of the ranking criterion proposed by Robins and Press and used in PACCMIT, it is shown that top ranking interactions correspond to more downregulated proteins than do the lower ranking interactions. Comparison with several other target prediction algorithms shows that the ranking of predictions provided by PACCMIT is at least as good as the ranking generated by other conservation-based methods and considerably better than the energy-based ranking used in other accessibility-based methods.

## Introduction

MicroRNAs (miRNAs) are endogenous small single stranded RNAs that modulate mRNA levels and/or translation in the cell. Recognition of the messenger by the miRNA is followed by either mRNA cleavage or translational repression, leading to a reduction in protein synthesis [1], [2]. Hundreds of targets involved in cell differentiation, development, cancer, cardiovascular disease, antiviral defense, and metabolism have been experimentally identified [1], [3], [4], [5], while thousands of genes are predicted to be under miRNA regulation in mammals [6]. For these reasons, uncovering the complex network of miRNA-mediated gene regulation plays a key role in understanding many biological processes taking place in the cell, and computational prediction of miRNA targets is an essential part of this challenge.

Due to the low cost of computational algorithms in comparison with the cost of experimental high throughput methods [7], [8], [9], , computational prediction of miRNA targets becomes increasingly popular for whole genome searches. Considerable effort has been devoted to developing bioinformatic tools with high precision and sensitivity [12], [13], [14], [15]. Since the most difficult task is achieving high precision [16], different methods try to reduce false positives by requiring long exact matches to the miRNA seed (i.e., 7 or 8 consecutive nucleotides in the 5′ end) [6], [17], [18], [19] or by demanding conservation [6], [18], [20], [21], [22], [23], [24], [25] or accessibility of the binding sites [17], [19], [23], [26], [27], [28], [29].

Comparative genomics has been used in miRNA target predictions since the first algorithms were proposed [24], [30]. Early observation of seed matches in conserved blocks of orthologous 3′UTRs in worms and flies [24] reinforced the assumption that looking for binding sites with conserved seed matches should increase the confidence in target predictions in animals in general. This assumption has been extrapolated successfully to herpes viruses. Murphy et al. [18] used conservation among viral strains in combination with over-representation of seed matches as a ranking criterion [25] to find functional targets of miRNAs in the human cytomegalovirus.

Methods considering the accessibility of the binding site instead of its conservation provided an alternative way of increasing precision [27]. Most of these methods rely less on the seed complementarity and more on the free energy differences [26], [27], [28], [29]. Although higher sensitivity is obtained by not rejecting binding sites with mismatches or wobble pairs in the seed region, precision is not always increased [19]. Other methods require perfect seed matches and employ different quantities than free energy differences to evaluate accessibility of the binding sites and to score predictions [17], [19]. One such method, PACMIT [19], uses the probability to find accessible stretches of four nucleotides in the seed matches instead of favorable free energy differences, and scores the likelihood of an interaction to be functional by estimating the over-representation of sites complementary to the seed. Ranking predictions by over-representation of seed matches results in a much higher precision than that obtained by other free-energy based methods. In addition, systematic comparisons in Ref. [19] showed explicitly that considering accessibility can in fact increase the precision of miRNA target predictions.

Although both conservation and accessibility restrictions improve the predictive power of miRNA target prediction [31], [32], [33], they are rarely used together in current methods. The first attempts to combine the two filters consisted in intersecting the predictions obtained using conservation-based methods with predictions obtained using accessibility-based methods [13], [34]. Although this procedure increased precision, it also drastically reduced sensitivity, suggesting that more efficient approaches to combine the two filters were needed. Several recent prediction algorithms consider both conservation and secondary structure of the target [15], [33], [35], however, these methods have been optimized for a single filter configuration that might not be the most appropriate in all biological contexts. For instance, since binding sites of highly conserved miRNAs are expected to be conserved, a conservation filter should be useful. On the other hand, the same reasoning might not apply for weakly conserved miRNAs. Due to the lack of flexibility of current methods, we know very little about how their performance is affected by using the two filters independently or together. Moreover, it is not clear if the performance of different filters depends on whether one predicts targets of highly or weakly conserved miRNAs.

Here we address these limitations by introducing a prediction method allowing the use of three different criteria (conservation, accessibility, or both) to filter putative binding sites while ranking the miRNA-3′UTR pairs according to the same score [25] in all three cases. The method, which we call PACCMIT (Prediction of ACcessible and/or Conserved MIcroRNA Targets), was obtained by generalizing the conservation filter from Refs. [18], [25] and integrating it into the accessibility-based method PACMIT. Effects that the three filters have on precision and sensitivity were compared on a dataset of validated targets obtained from photoactivatable-ribonucleoside-enhanced crosslinking and immunoprecipitation (PAR-CLIP) experiments [9]. Our results show that while the conservation filter is more effective than the accessibility filter to predict targets of highly conserved miRNAs, accessibility performs better than conservation in the case of weakly conserved miRNAs. Moreover, in the case of highly conserved microRNAs, the performance was improved even further, especially among the top predictions, by using the combined filter. For reference, we compare results of PACCMIT and nine other target prediction methods, and show that the ranking of targets obtained with PACCMIT is not only consistent with the downregulation of protein levels of targets but also comparable to or better than the ranking obtained with other available target prediction tools.

## Methods

### 3′UTR sequence alignments and miRNA sequences

Genomic coordinates of Ensembl human genes (hg18) were used to extract the human 3′UTR sequences and the corresponding aligned sequences from the 28-species alignment (MAF file) available at the UCSC Table browser (http://genome.ucsc.edu) [36], [37]. Only protein coding genes were included in the database and when several mRNA isoforms were reported for the same Ensembl gene ID, only the one with the longest 3′UTR sequence was used in the analysis. Mature human miRNA sequences were obtained from the miRBase website (http://www.mirbase.org) [38]. Following the classification on TargetScan website (http://www.targetscan.org) [6], miRNAs are considered to be conserved if they share the same seed sequence (positions 2–8) in different species. Specifically, miRNAs are said to be ‘highly conserved’ if they are classified as such in TargetScan classification. On the other hand, miRNAs considered as ‘conserved’ and ‘poorly conserved’ in TargetScan classification are grouped in a single set of ‘weakly conserved’ miRNAs.

### Training dataset

In order to find an optimal choice for the conservation filter in PACCMIT we used the proteomics dataset reported by Baek et al. [8]. This dataset covers three highly conserved miRNAs. For the sake of statistical analysis, an arbitrary classification of the miRNA-gene pairs between functional and non-functional was performed based on the log_2_ fold changes (log_2_FC) in protein expression, with the same cutoff as in other studies [8], [13], [19]. Specifically, miRNA-gene pairs with log_2_FC≤−0.2 were labeled as functional targets while the remaining pairs were labeled as non-functional targets.

### Test datasets

In order to test the effect of different filters on sensitivity and precision of target predictions for highly and weakly conserved miRNAs, we constructed positive and negative datasets using the binding sites reported in the PAR-CLIP experiments [9]. Similarly as the authors of the PAR-CLIP paper, we only focused on the 100 most abundant miRNAs since these account for 96% of the miRNA sequence reads. The set of 100 most abundant miRNAs was divided into two groups containing 74 highly conserved and 26 weakly conserved miRNAs (Table S1). In each group, functional miRNA-gene pairs were defined as those pairs in which at least one 7-mer matching miRNA positions 2–8 was found between positions 21 and 30 of the cluster-centered regions (CCR) that were mapped to the 3′UTRs (human assembly hg18). This particular location in the CCR was used because according to the PAR-CLIP validation, a majority of the perfect miRNA seed matches are found in that region of the CCR [9].

As for the negative datasets, we first selected all unbound genes, i.e., all genes for which no CCR could be mapped to any region of the whole transcript. Then, for each group of miRNAs (i.e., for either highly or weakly conserved miRNAs), we generated all possible combinations between the miRNAs and the unbound genes. Finally, the negative datasets of non-functional pairs were constructed by randomly selecting *N* pairs from the previously generated combinations, where *N* was chosen to be equal to the number of functional pairs found for the same group of miRNAs. Thus, the number of generated non-functional pairs was equal to the number of functional pairs found for each group. We intentionally constructed the negative dataset of the same size as the positive dataset in order that the values of precision achieved by various methods were well distributed between 0 and 1 and not concentrated at either of the extremes, as could happen if the proportion of negatives in the dataset were too high or too low. In the case of highly conserved miRNAs, *N* = 3,586, while in the case of weakly conserved miRNAs, *N* = 112. We called these the ‘large’ datasets.

On average, only 0.6 miRNAs are matching a given CCR in the indicated positions (if we consider all the CCR regions in the 3′UTRs). If we only consider the CCRs that contain at least one seed match, this number increases to 3.1 miRNAs per CCR.

In order to analyze the statistical significance of the precision and sensitivity values of the different methods, each dataset of 2*N* validated pairs (*N* functional and *N* non-functional) was further divided into three smaller sets that we refer to as ‘small’ datasets. This partition was done by dividing each group of miRNAs into three subgroups of similar size and dividing the ‘large’ dataset accordingly. The subgroups of highly conserved miRNAs contained 25, 25, and 24 miRNAs. The subgroups of weakly conserved miRNAs contained 9, 9, and 8 miRNAs. Number of ‘*targets per miRNA*’ and ‘*precision*’, which are discussed in the Results section and displayed in the figures below, are the mean values obtained by averaging over the three ‘small’ datasets. The corresponding error bars are the standard errors of the mean obtained from the three ‘small’ datasets. Statistical significance of the difference between various methods was evaluated with the one-sided *t* test. The *P*-values can be found in Tables S3 and S4.

To evaluate the quality of the ranking of targets by PACCMIT, we used the proteomics data of Selbach et al. [7] which provides the protein log_2_ fold changes measured after over-expression of five highly conserved miRNAs.

### Computation of accessibility in PACCMIT

Accessibility was evaluated in the same way as in Ref. [19]. Any 7-mer in the 3′UTR sequence (including seed matches) was catalogued as accessible if it contained at least one 4-mer unpaired with a probability *P*_free_≥*P*_cutoff_, where *P*_cutoff_ had an optimized value of 0.2. Calculation of *P*_free_ values for all 4-mers in all the human 3′UTR sequences was performed with the program RNAplfold [39] using a window *W* = 80 and a maximum pairing distance *L* = 40 as recommended in Ref. [40].

### Scoring of miRNA-3′UTR interactions in PACCMIT

The list of predicted miRNA-3′UTR interactions is always ranked according to the single hypothesis *P* value (*P*_SH_), which is a statistical score capable to account simultaneously for single and multiple binding sites as well as to accommodate accessibility and/or conservation filters. *P*_SH_ is an approximate probability that a given oligomer (e.g., a 7-mer), complementary to the miRNA seed, is found by chance at least *c* times in the corresponding 3′UTR. Lower values of *P*_SH_ imply that the interaction is more likely to be functional (see Refs. [18], [19] for details about *P*_SH_). *P*_SH_ is computed as
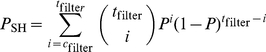
(1)where *t*_filter_ and *c*_filter_ are respectively the total number of 7-mers in a 3′UTR sequence that meet the filter requirement and the number of seed matches that meet the filter requirement. The possible ‘filters’ are: ‘access’ (i.e., only accessible 7-mers are counted), ‘cons’ (i.e., only conserved 7-mers are counted), or ‘cons+access’ (i.e., only 7-mers that are both conserved and accessible are counted). If no filter is specified, the whole 3′UTR and all seed matches are considered. All results shown in this work were obtained using 7-mers matching (i.e., complementary to) the miRNA positions 2–8. However the algorithm allows looking for shorter or longer matches and also for matches with varying starting position.

### Predictions of other methods for human miRNAs

Results of DIANA-microT v3.0 [21], PicTar [20], ElMMo v5 [22], TargetScan 5.2 [6], [41], miRanda [42], MirSVR [35], PITA [27], and IntaRNA [29] were used for comparison with PACCMIT. The details for each method are: i) DIANA-microT v3.0: bulk data were downloaded from http://diana.cslab.ece.ntua.gr/microT; predictions were ranked by ‘miTG score’; only predictions above a cutoff of 7.3 were considered as suggested by the authors. ii) PicTar: bulk data were downloaded from the UCSC browser (in July 2010) as explained in http://pictar.mdc-berlin.de; predictions were ranked by the scaled ‘PicTar score’; conservation in four species (human, mouse, rat, and dog) was used. iii) ElMMo v5: bulk data were downloaded from http://www.mirz.unibas.ch/miRNAtargetPredictionBulk.php; predictions were ranked according to the probability ‘p’ that the site is under evolutionary selective pressure; only predictions above a cutoff of 0.5 were considered as recommended by the authors. iv) TargetScan 5.2: the list of ‘Summary Counts’ was downloaded from http://www.targetscan.org; predictions were ranked according to the aggregate *P*_CT_ score recommended by the authors to assess the biological relevance of the predicted interaction and also according to the total context score. *P*_CT_ score was only available for the set of highly conserved miRNAs. v) miRanda: the version of the software from August 2010 was downloaded from http://www.microrna.org/microrna/getDownloads.do; targets were predicted using default parameters and ranked according to the total score. vi) MirSVR: bulk data (released in August 2010) for conserved and non-conserved miRNAs were downloaded from http://www.microrna.org/microrna/getDownloads.do; predictions were ranked according to the sum of the scores for individual sites as recommended by the authors. vii) PITA: the first and only public version of the software was obtained from http://genie.weizmann.ac.il/pubs/mir07/mir07_prediction.html; targets were predicted using default parameters and ranked according to the PITA score. viii) IntaRNA 1.2.2: the software was downloaded from http://www.bioinf.uni-freiburg.de/Software; targets were predicted using the seed 2–8, w = 80 and L = 40. Predictions were ranked by optimal energy score. In order to compare with the experimental datasets, gene names in the predictions of other methods were translated from RefSeq IDs, gene symbols, or gene IDs to Ensembl gene IDs using the BioMart tool corresponding to Ensembl54 (available at http://may2009.archive.ensembl.org) [43].

## Results

### Two different approaches to filter seed matches by conservation

Different target prediction methods have implemented different approaches to filter seed matches according to their conservation [6], [18], [20], [21], [22]. The degree of conservation of the binding site is generally judged by the number of species with the same sequence and/or by the phylogenetic distance between the species sharing the same sequence. Motivated by these two main strategies, we designed two simple approaches to judge a site as conserved (see Figure S1): (i) in the “Any-species” (Any-*S*) approach, the seed match must be present in the aligned sequences of at least *S* species (including the human), regardless of their distance from the human. Increasing *S* makes the conservation filter more stringent. (ii) In the “Selected-species” (Selected-*S*) approach, the seed match must be present in the aligned sequences of specific *S* species. The stringency is again increased by increasing *S*, but now the (*S*+1)^st^ added species is pre-selected and is more distant from the human than the preceding *S* species. In this approach, we only included those species in which the seeds of the eight miRNAs from the proteomics datasets were conserved (see Figure S1). The conservation filter was optimized using a training dataset constructed from the proteomics data of Baek et al. [8] (see Methods). Site conservation was obtained from the 28-species alignment available at the UCSC Table Browser [37] and from the topology of the phylogenetic tree reported by Miller et al. [36].

**Figure 1:**
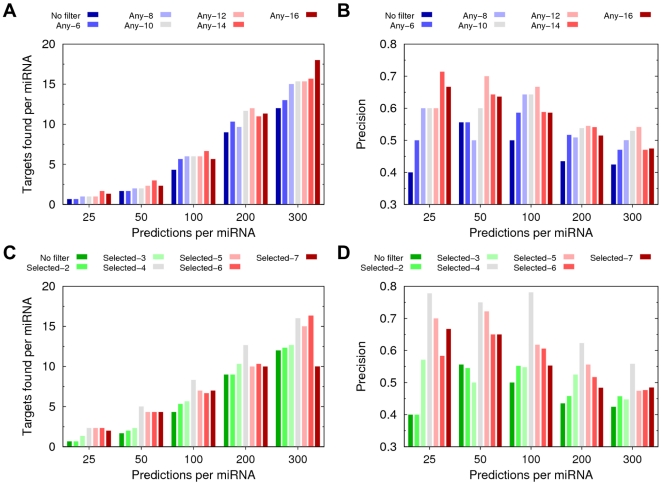
Determination of the optimal conservation filter in PACCMIT. (**A–B**) Number of validated targets per miRNA (panel **A**) and precision (panel **B**) of the top predictions of PACCMIT using different number *S* of species in the “Any-species” approach. (**C–D**) The same as in panels (**A**–**B**) but using the “Selected-species” approach. The definitions of different levels of stringency of the conservation filter are: Selected-2 = human and chimp, Selected-3 = Selected-2 and rhesus, Selected-4 = Selected-3 and mouse, Selected-5 = Selected-4 and dog, Selected-6 = Selected-5 and cow, and Selected-7 = Selected-6 and chicken. In all four panels, the number of predictions on the horizontal axis is normalized by the number of miRNAs, i.e., it is displayed as “predictions per miRNA.” Note that in the case of Selected-7 (and only in this case) fewer than 300 predictions per miRNA were obtained, which explains the abrupt decrease in the number of targets per miRNA found among the top 300 predictions per miRNA in panel (**C**).

Precision and the number of true targets per miRNA were computed as functions of the number of predictions per miRNA for varying stringency of the conservation filter (Figure 1). For both approaches and for all levels of stringency of the conservation filter, PACCMIT performed better with the conservation filter than without it. Note, however, that, regardless of the approach used, the most restrictive configuration was not necessarily the optimal choice. For instance, in the Any-species approach, only small fluctuations were observed in the number of targets per miRNA found among the top 25, 50, and 100 predictions per miRNA for different levels of stringency. It was only among the 200 and 300 predictions per miRNA that using more species in the filter helped recovering more validated targets (Figure 1A). As for precision, when the statistics become sufficient (i.e., for 50 or more predictions per miRNA), the best overall performance appears to be for *S* = 12 species (Figure 1B). On the other hand, among different configurations of the Selected-species approach, we found Selected-4 to be clearly optimal as it showed both the best sensitivity (Figure 1C) and the best precision (Figure 1D) among the top predictions per miRNA. Given that the conservation filter with four selected species performs better than the filter with any twelve species (compare Figure 1C–D with Figure 1A–B), the former has been used for all further analyses, unless specifically indicated otherwise.

### Accessibility is a better filter than conservation when predicting targets of weakly conserved miRNAs

There is abundant evidence (also corroborated by our results on the training dataset) confirming the relevance of the site conservation requirement in miRNA target prediction. This is not surprising since this requirement is based on the assumption that highly conserved miRNAs should have highly conserved binding sites in order to maintain miRNA function. However, this reasoning may not apply to other miRNAs that are either weakly conserved or that are not conserved at all. In such cases, using a conservation filter may not be particularly useful. In order to investigate the differences between these two scenarios, we applied PACCMIT with various filters to two datasets of experimentally validated targets: the former dataset consisted of targets of highly conserved miRNAs while the latter was composed of targets of weakly conserved miRNAs (see Methods).

**Figure 2:**
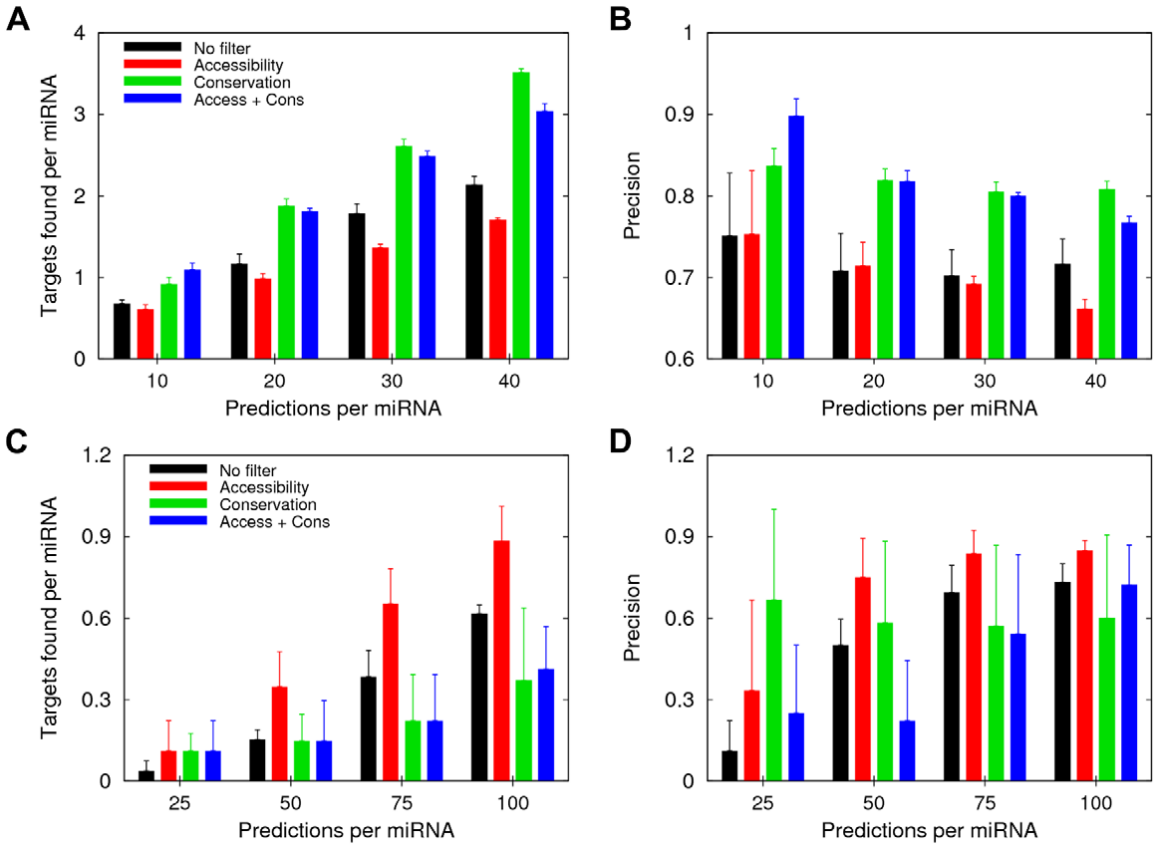
Accessibility is a better filter than conservation when predicting targets of weakly conserved miRNAs. (**A**–**B**) Number of validated targets per miRNA (panel **A**) and precision (panel **B**) of the top predictions of PACCMIT for highly conserved miRNAs. Error bars represent the standard errors of the mean computed from three ‘small’ datasets (see Methods). Results of PACCMIT without any filter (“No filter”), with single filters (“Accessibility” or “Conservation”), and with the combined filter (“Access+Cons”) are shown in both panels. (**C**–**D**) The same as in panels (**A**–**B**) but for weakly conserved miRNAs.

We have found that for highly conserved miRNAs, the conservation filter provides more true targets per miRNA and higher precision than does the accessibility filter (Figure 2A–B), and that the differences are statistically significant according to the one-sided *t* test (see Table S3 for the *P*-values). The same behavior is observed in the corresponding precision vs. sensitivity plots in which the curve for PACCMIT ‘Cons’ lies above the curves of both PACCMIT ‘No filter’ and PACCMIT ‘Access’ (Figure S2A). This is consistent with the results from two previous studies [31], [32] in which site conservation was found to be a better predictor of miRNA targets than site accessibility. However, in the case of weakly conserved miRNAs a completely opposite situation emerges (Figures 2C–D and S2B). Here the conservation filter is outperformed not only by the accessibility filter but in many cases also by the algorithm with no filter at all. While the superiority of the accessibility filter is less statistically significant than the superiority of conservation was for highly conserved miRNAs (see Table S3), Figure 2C–D shows clearly that conservation is not an appropriate filter for weakly conserved miRNAs. At first glance, the behavior exposed in Figure 2C–D might seem in conflict with the results from Wen et al. [32], who reported a higher predictive power for seed conservation than for site accessibility on a PAR-CLIP dataset of 20 miRNAs. However, since 17 out of the 20 miRNAs selected for that study were highly conserved, conclusions of Wen et al. cannot be automatically extended to weakly conserved miRNAs. In fact, in the same study, seed conservation showed higher predictive power than accessibility only for one out of the three weakly conserved miRNAs. Similar caution should be taken when interpreting results obtained from the transcriptomics and proteomics datasets in Refs. [31], [32] since most of the miRNAs over- or under-expressed in those experiments were highly conserved. Altogether, our findings suggest that using a conservation filter is critical only when predicting targets for highly conserved miRNAs. In order to predict targets for weakly conserved miRNAs other criteria should be considered, and our results show that accessibility is one of them.

**Figure 3:**
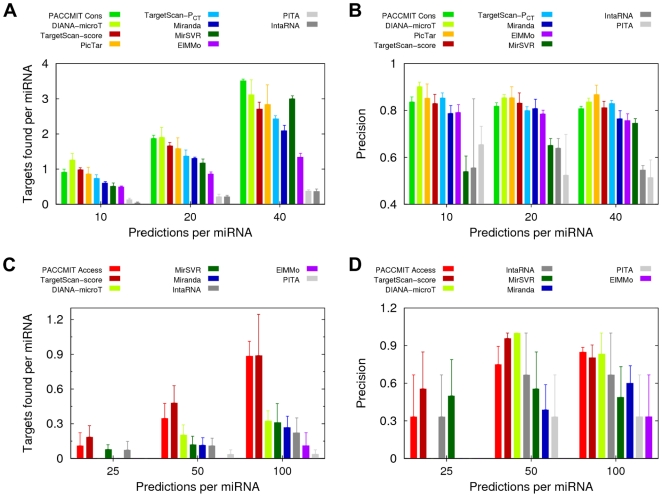
Comparison of sensitivity and precision of various methods in predicting targets of highly and weakly conserved miRNAs. (**A**–**B**) Number of validated targets per miRNA (panel **A**) and precision (panel **B**) of the top predictions of different methods for highly conserved miRNAs. (**C**–**D**) The same as in panels (**A**–**B**) but for weakly conserved miRNAs. Results of PACCMIT with the conservation filter (panels **A**–**B**) and with the accessibility filter (panels **C**–**D**) are included for comparison. TargetScan predictions ranked by context score are labeled as “TargetScan-score” while its predictions ranked by probability *P*_CT_ are labeled as “TargetScan-P_CT_”. [The latter are not shown in panels (**C**–**D**) because *P*_CT_ is not available for targets of weakly conserved miRNAs.] Given that available predictions of PicTar involve only 3 of the 26 weakly conserved miRNAs, we did not consider this method for this part of the analysis. In panel (**D**), precision of the top 25 predictions per miRNA is not shown for DIANA-microT, Miranda, ElMMo, and PITA because precision is not defined for these methods, i.e., there are no true or false positives found yet. For details about the version and/or release date of each prediction method see the Methods section.

Figure 3 shows results of nine available prediction methods in the two situations analyzed above. Some of those methods are based on site conservation (DIANA-microT [21], TargetScan-P_CT_ [6], PicTar [20], and ElMMo [22]), while others rely on site accessibility (PITA [27], IntaRNA [29]) and one on both criteria (MirSVR [35]). In each case, results of PACCMIT using the most appropriate filter (i.e., either conservation or accessibility) are shown for reference (see Table S4 for the *P*-values of the comparison between those methods and PACCMIT). The figure demonstrates that when predicting targets for highly conserved miRNAs, conservation-based methods perform much better than methods based on site accessibility (Figure 3A–B). On the other hand, in the case of weakly conserved miRNAs, conservation-based methods are not better than those based on site accessibility (Figure 3C–D). Remarkably, the two methods performing the best in this scenario, at least with respect to the number of targets found per miRNA (i.e., “PACCMIT Access” and “TargetScan-score”), do not consider conservation of the binding sites at all; “PACCMIT Access” considers all accessible seed matches while “TargetScan-score” scores binding sites according to the local A/U content, additional 3′ pairing, and relative position in the 3′UTR (these three criteria being grouped into the so-called ‘context score’).

Given the *simplicity* of our conservation and accessibility filters, we found remarkable that PACCMIT emerged among the best performing methods in both scenarios. Here, by *simplicity* we mean that accessibility and conservation are only used as restrictions to discard some seed matches rather than as scores to rank the miRNA-3′UTR interactions, which is done in more sophisticated approaches. For instance, in the case of accessibility, PITA and IntaRNA use the differences between the so-called opening and hybridization energies to rank the interactions. Similarly, most conservation-based methods use the degree of conservation to rank the miRNA-3′UTR pairs.

As the reader may have inferred from Figures 2 and 3, highly conserved miRNAs have more targets per miRNA among the top predictions than the weakly conserved miRNAs; overall, we found 48.5 targets per highly conserved miRNA and only 4.3 targets per weakly conserved miRNA. This difference can be justified by the fact that highly conserved miRNAs are more likely to have accumulated more targets throughout evolution.

### Combination of conservation and accessibility filters in PACCMIT improves the predictions of highly conserved miRNAs

**Figure 4:**
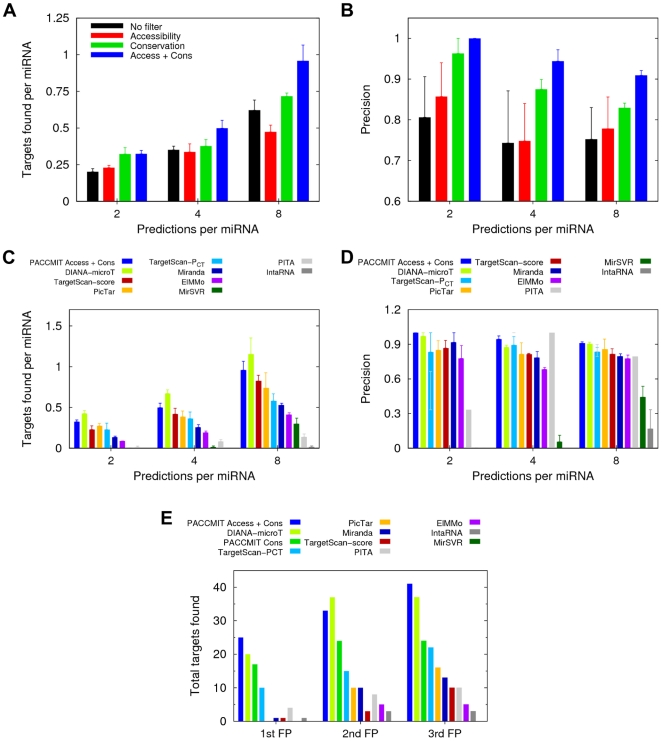
Combination of conservation and accessibility can improve performance over individual filters. (**A**–**B**) Number of validated targets per miRNA (**A**) and precision (**B**) of the topmost predictions of PACCMIT for highly conserved miRNAs. (**C**–**D**) Comparison of PACCMIT (using the combined filter) with different methods under the same conditions as in panels (**A**–**B**). In panel (**D**), the precision of the top two predictions per miRNA is not defined for MirSVR. (**E**) Number of validated targets found before the first, second, and third false positive appears in the ranked predictions for highly conserved miRNAs. For this analysis we used the ‘large’ dataset of validated targets that involve all 74 highly conserved miRNAs (see Methods).

Assuming that the binding sites of highly conserved miRNAs should be *both* conserved *and* accessible, one would expect the combined filter to outperform the single filters. Although that turned out not to be the case when evaluating 20 or more predictions per miRNA, for 10 predictions per miRNA the combined filter indeed performed slightly better than the conservation filter (Figure 2A–B). This suggested that the double restriction could outperform the single filters more markedly among the very top predictions, which was confirmed by focusing our analysis on eight or fewer predictions per miRNA (see Figure 4A–B and *P*-values in Table S3). The same behavior can be seen in Figure S2A where at low sensitivity the combined filter shows higher precision than either filter alone. Comparison with other available methods confirmed that PACCMIT belonged again among the most competitive methods; it was outperformed only by DIANA-microT (Figure 4C–D).

The conservation and combined filters used in PACCMIT were further compared by measuring the number of true targets obtained before the first, second, and third false positive (i.e., non-functional pair) appeared in the predictions. Higher numbers of true targets were always obtained when *both* conservation *and* accessibility were required, implying that some non-functional yet conserved seed matches could be successfully rejected with the accessibility criterion (Figure 4E). The results of other methods are shown for reference.

**Figure 5:**
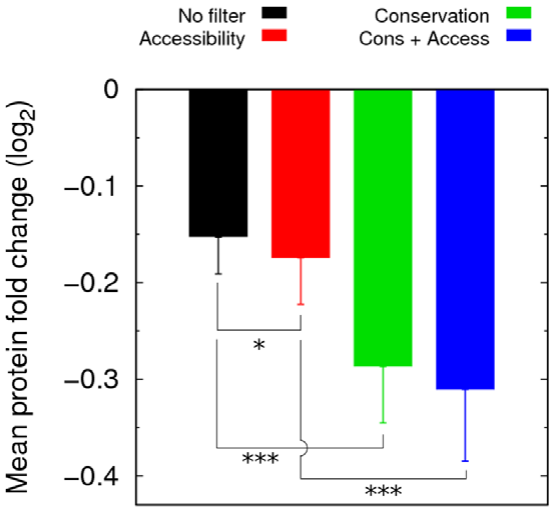
Predictions filtered by conservation, accessibility, or both are more downregulated than non-filtered predictions. Mean log_2_ fold changes in protein expression are shown for the targets of five highly conserved miRNAs, predicted using PACCMIT with different filters. The log_2_ fold changes are taken from Selbach et al. [7]. Statistical significance is given by the one-sided Wilcoxon rank sum test (*P≤0.05, **P≤0.01, ***P≤0.001). *P*-values>0.05 are not indicated. Error bars indicate standard errors of the mean.

In order to see if the improvements obtained with the different filters were also reflected in the downregulation of the targets, we computed the mean log_2_ fold changes (log_2_FC) in protein expression of miRNA targets predicted with different configurations of PACCMIT. For this purpose, we used the proteomics data from Selbach et al. [7]. Figure 5 shows that the mean log_2_FC in protein expression is indeed more negative for targets predicted with a single filter than for targets predicted with no filter. Moreover, the mean log_2_FC is the most negative for genes predicted with the combined filter, although only the difference with respect to the accessibility filter was statistically significant. It is not surprising that the conservation filter performs considerably better than the accessibility filter, given that the five miRNAs in this dataset are highly conserved.

Altogether, results presented in Figures 4, 5, and S2A show that the use of the combined filter can improve sensitivity, precision, and quality (measured by downregulation of targets) of the top predictions for highly conserved miRNAs in comparison with the use of each filter separately. The figures also reaffirm that in that scenario the conservation filter is more effective than the accessibility filter.

As far as ranking predictions is concerned, when both filters are used simultaneously, *P*_SH_ is computed using Eq. (1) (see Methods), taking *t*_filter_ equal to the total number *t*_cons+access_ of 7-mers in the 3′UTR that are both conserved and accessible (regardless of their complementarity to the seed) and *c*_filter_ equal to the number *c*_cons+access_ of conserved and accessible seed matches.

### Ranking predictions according to *P*_SH_ is correlated with the extent of target downregulation

**Figure 6:**
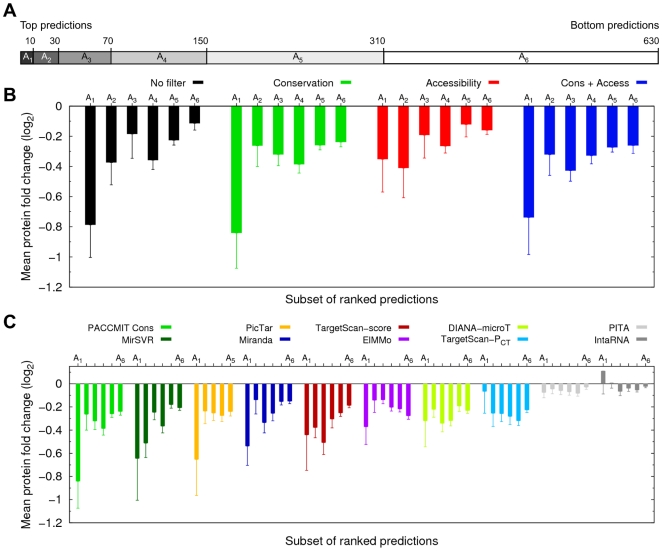
Top predictions ranked according to *P*_SH_ correspond to strongly downregulated targets. (**A**) Predictions of PACCMIT analyzed in Figure 5 are partitioned into non-overlapping subsets A_j_ of increasing size. The first subset (A_1_) contains the top 10 predictions, the second subset (A_2_) contains the next 20 predictions (11th to 30th), the third subset (A_3_) contains the next 40 predictions (31st to 70th), etc. (**B**) The mean log_2_ fold changes in protein expression are shown for each subset of ranked predictions and the four possible filter configurations of PACCMIT are compared. (**C**) The same analysis as in panel (**B**) is applied to nine standard target prediction methods. Results of “PACCMIT Cons” are included for comparison. Error bars indicate standard errors of the mean.

Analysis of the log_2_FC in target expression, presented in the previous section, illustrates the quality of different filters, but says nothing about the quality of the ranking of predictions. In other words, we cannot tell from Figure 5 whether the top ranked predictions correspond to proteins that are more, equally, or less downregulated than those among the bottom predictions. To answer this question we partitioned the predictions for the five miRNAs in the Selbach dataset into several non-overlapping subsets of increasing size (see Figure 6A) and computed the mean log_2_FC for each subset. Our results showed that both in the presence and in the absence of a filter, the top ranked targets have a more negative mean log_2_FC than the rest of the targets in the list (Figure 6B).

When different algorithms are compared in the literature, sometimes the ranking is ignored and only the overall sensitivity and precision are considered (we show these in Table S2). Similarly to Figure 5, such comparisons only evaluate the filters (or cutoff values for different quantities) used in different algorithms that can be tuned at will. However, until a perfect algorithm is found, ranking of predictions will be very important in order to guide experiments. In Ref. [19] a detailed analysis of several ranking criteria showed that over-representation, measured by *P*_SH_, is more successful in ranking predictions than other scores such as the hybridization or total free energies. Analysis in Ref. [19] was based on a binary classification of predictions as true or false positives. Here we took a more quantitative approach, finding that the ranking according to *P*_SH_, used in PACCMIT, also correlates with the degree of protein repression expected from a predicted miRNA-gene interaction. A qualitative comparison of the ranking obtained with different methods shows that *P*_SH_ is among the best ranking criteria (Figure 6C). The same comparison also confirms that scoring miRNA-3′UTR interactions according to the thermodynamic stability of the pairing along the whole miRNA (as is done, e.g., in PITA and IntaRNA) does not necessarily reflect their biological functionality, as has been already argued by Robins et al. [17] and by Marin and Vanicek [19]. Analysis similar to that presented in Figure 6, but in which the predictions were partitioned into bins of the same instead of increasing size, led to the same conclusions as those drawn from Figure 6 (see Figure S3).

## Discussion

Although conservation and accessibility are known to be important factors reducing the false discovery rate in target prediction methods, they are not usually exploited in a combined and flexible manner. Here we have used the statistical framework introduced by Robins and Press [25] to develop PACCMIT, an miRNA target prediction method capable of filtering putative binding sites according to their accessibility and/or conservation. The first application of PACCMIT has revealed that although conservation is the most appropriate filter for predicting targets of highly conserved miRNAs, it is not equally effective in predicting targets of weakly conserved miRNAs. For those miRNAs, target site accessibility turns out to be the more appropriate filter. Moreover, in the case of highly conserved miRNAs we have found that a combined filter is more effective in discarding false positives than either the conservation or accessibility filters alone. Additional comparisons between PACCMIT and nine standard prediction methods confirmed the advantages of using the conservation filter in predicting targets of highly conserved miRNAs. These comparisons also showed that target prediction for weakly conserved miRNAs cannot rely on site conservation as heavily as it does in most available prediction tools. Therefore, it is important to identify other criteria that would be as useful as accessibility in the prediction of targets for non-conserved miRNAs.

When performing genome-wide target searches, it is desirable to have methods with low false discovery rates in order to avoid extensive lists of low confidence predictions. Since designing such methods is much more difficult than generating methods with high or even perfect sensitivity, our efforts have been directed more towards increasing precision than covering all known miRNA targets. However, PACCMIT's sensitivity can be expanded thanks to the possibility to adapt its search according to the information available. Requiring only accessibility allows searching for species-specific targets (which are not expected to have conserved binding sites) and for targets in genomes for which conservation information is difficult to obtain. Sensitivity of PACCMIT can be also modulated easily by using different stringency levels in the conservation or accessibility filters. This flexibility is due to the simple underlying statistical framework of PACCMIT, providing a single scoring function (*P*_SH_) that can easily accommodate various filters (as explained in the Methods section). However, *P*_SH_ is more than a score to rank the predictions; it is also a statistical estimate that the predicted interaction occurs by chance (lower *P*_SH_ values imply a higher likelihood that the predicted interaction is functional). It has been shown previously that *P*_SH_ is a better ranking criterion than several free energy scores [19]. Here we also show that ranking by *P*_SH_ is in good agreement with the protein fold changes: in comparison with lower ranking interactions, top ranking interactions correspond to more downregulated genes. This observation holds regardless of which filter configuration of PACCMIT is used. We have shown that *P*_SH_ is at least as good as other types of scores implemented in conservation-based methods and considerably better than the energy-based scores implemented in other accessibility-based methods.

The miRNA target predictions of PACCMIT in the human using different filter configurations can be found at http://lcpt.epfl.ch.

## Supporting Information

Figure S1 **Tree topology of the 28-species alignment used to compute conservation.** The two approaches used in PACCMIT to filter sites by conservation are illustrated with examples: In the “Any-species” approach, we show one possible configuration of PACCMIT with *S* = 8 (i.e., Any-8). In the “Selected-species” approach, all six possibilities studied here are shown, i.e., Selected-*S* for *S* = 2, 3, 4, 5, 6, and 7.
(TIFF)

Figure S2 **Precision as a function of sensitivity using different filter configurations of PACCMIT.** (**A**) Precision of PACCMIT plotted as a function of sensitivity for predicted targets of highly conserved miRNAs. (**B**) The same as in panel (**A**) but for weakly conserved miRNAs.
(TIFF)

Figure S3 **Correlation between ranking and target downreguation for different miRNA target prediction methods.** (**A**) The mean log_2_ fold changes are shown for each subset of ranked predictions for the different filter configurations of PACCMIT. Each bin represents 100 predictions: A_1_: top 100 predictions, A_2_: predictions 101 to 200, A_3_: predictions 201 to 300, and A_4_: predictions 301 to 400. (**B**) Similar analysis as in panel (**A**) is applied to nine standard prediction methods. The results of “PACCMIT Cons” are also included for comparison. Error bars indicate standard errors of the mean.
(TIFF)

Table S1 **List of the highly and weakly conserved miRNAs used in this study.**
(DOC)

Table S2 **Overall sensitivity and precision obtained with PACCMIT and other methods.**
(DOC)

Table S3 **Statistical significance of the differences between the four configurations of PACCMIT.** *P*-values were obtained from a one-sided *t* test and correspond to the null hypothesis.
(DOC)

Table S4 **Statistical significance of the differences between PACCMIT and other methods.** *P*-values were obtained from a one-sided *t* test and correspond to the null hypothesis.
(DOC)
