# Supplementary figures and images for: Optimal Use of Conservation and Accessibility Filters in MicroRNA Target Prediction
Source: PLoS One. 2012 Feb 27;7(2):e32208. doi: 10.1371/journal.pone.0032208 (PMC3288066; doi:10.1371/journal.pone.0032208)

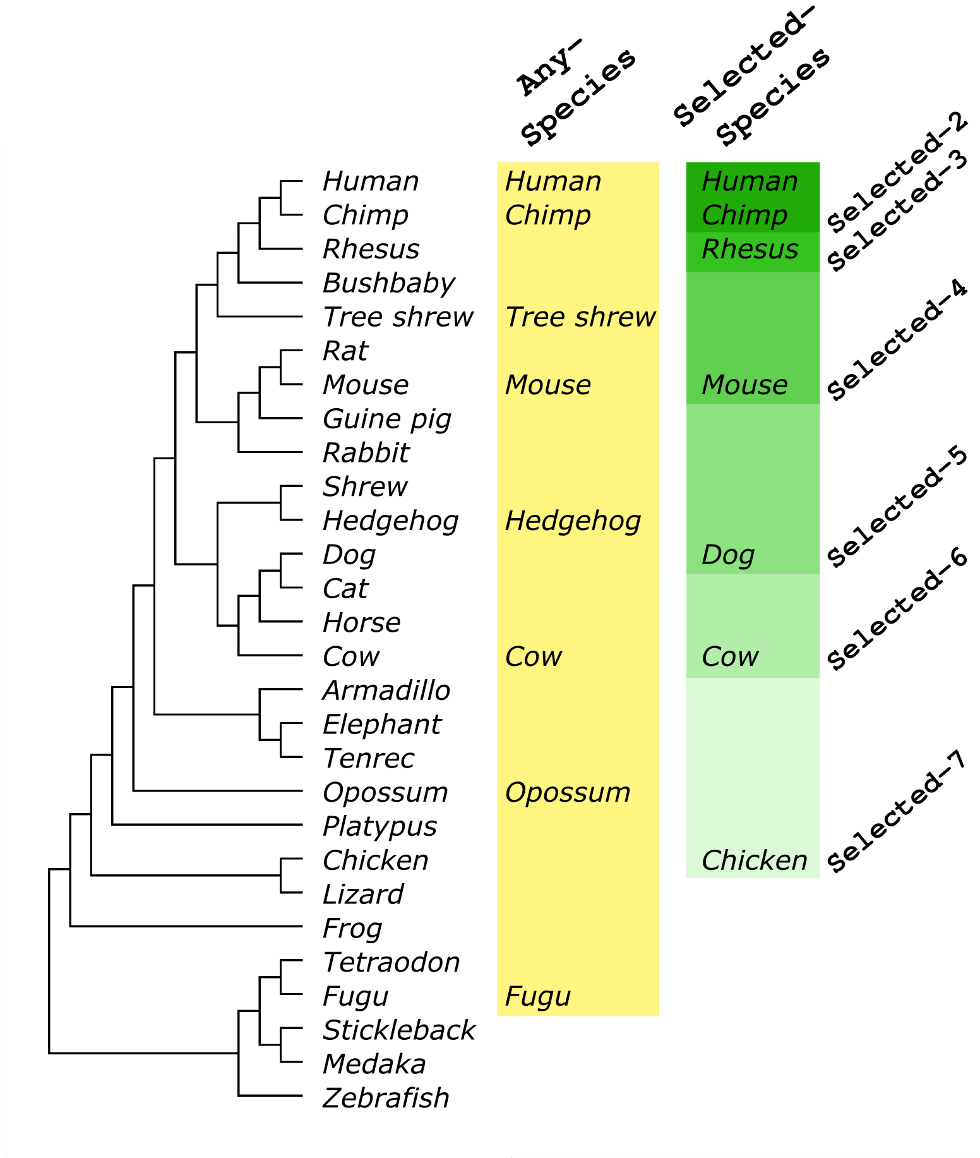

Supplement: Figure S1 — Tree topology of the 28-species alignment used to compute conservation. The two approaches used in PACCMIT to filter sites by conservation are illustrated with examples: In the “Any-species” approach, we show one possible configuration of PACCMIT with S = 8 (i.e., Any-8). In the “Selected-species” approach, all six possibilities studied here are shown, i.e., Selected-S for S = 2, 3, 4, 5, 6, and 7. (TIFF) [file pone.0032208.s001.tif]

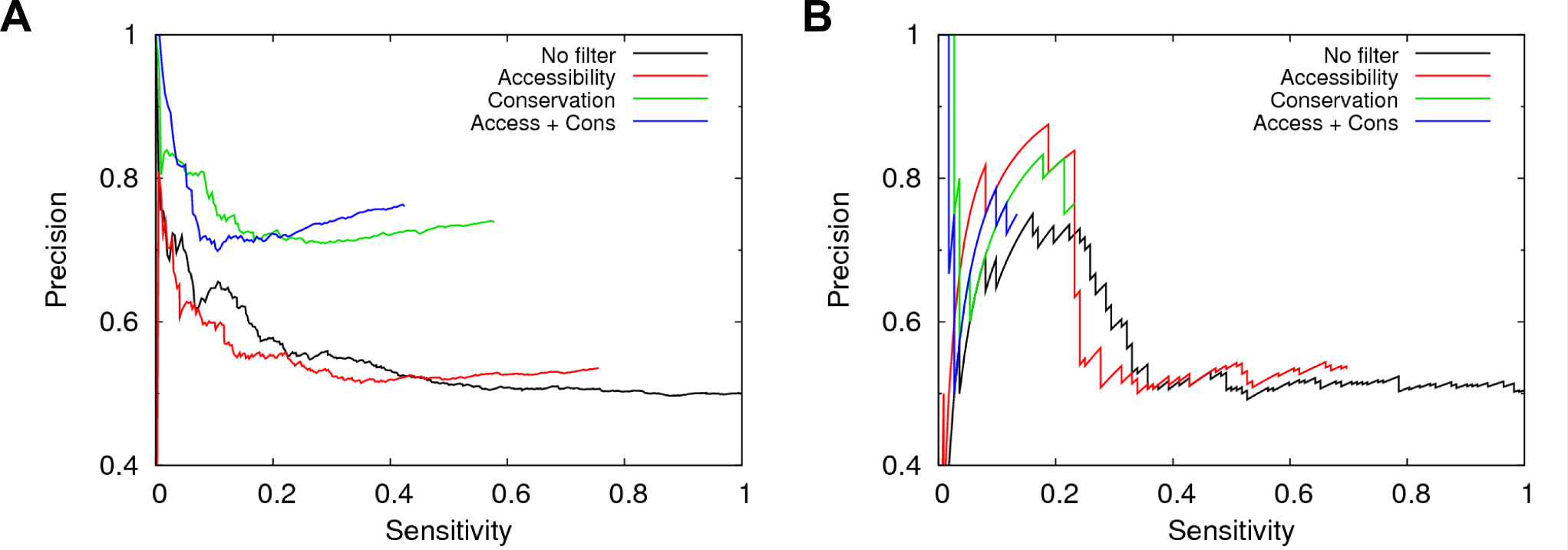

Supplement: Figure S2 — Precision as a function of sensitivity using different filter configurations of PACCMIT. (A) Precision of PACCMIT plotted as a function of sensitivity for predicted targets of highly conserved miRNAs. (B) The same as in panel (A) but for weakly conserved miRNAs. (TIFF) [file pone.0032208.s002.tif]

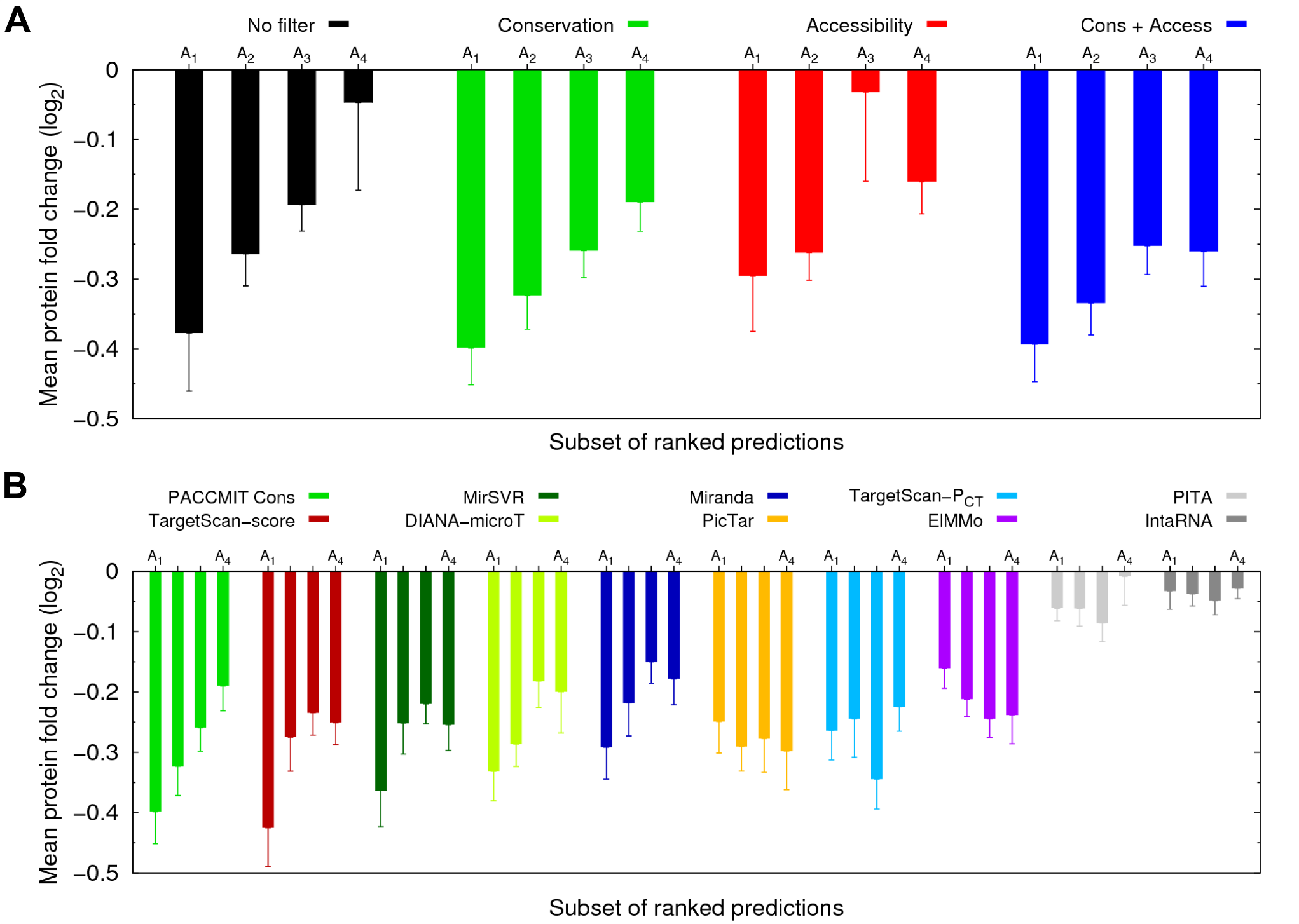

Supplement: Figure S3 — Correlation between ranking and target downreguation for different miRNA target prediction methods. (A) The mean log2 fold changes are shown for each subset of ranked predictions for the different filter configurations of PACCMIT. Each bin represents 100 predictions: A1: top 100 predictions, A2: predictions 101 to 200, A3: predictions 201 to 300, and A4: predictions 301 to 400. (B) Similar analysis as in panel (A) is applied to nine standard prediction methods. The results of “PACCMIT Cons” are also included for comparison. Error bars indicate standard errors of the mean. (TIFF) [file pone.0032208.s003.tif]
