# Supplementary material for: Optimal Use of Conservation and Accessibility Filters in MicroRNA Target Prediction
Source: PLoS One. 2012 Feb 27;7(2):e32208. doi: 10.1371/journal.pone.0032208 (PMC3288066; doi:10.1371/journal.pone.0032208)
Supplement: Table S1 — List of the highly and weakly conserved miRNAs used in this study. (DOC) [file pone.0032208.s004.doc]

Table S1. List of the highly and weakly conserved miRNAs used in this study.

| **Highly conserved miRNAs** | | | | | **Weakly conserved miRNAs** | |
| --- | --- | --- | --- | --- | --- | --- |
| hsa-let-7a | hsa-miR-17 | hsa-miR-29b | hsa-miR-101 | hsa-miR-183 | hsa-miR-140-3p | hsa-miR-423-3p |
| hsa-let-7b | hsa-miR-18a | hsa-miR-29c | hsa-miR-103 | hsa-miR-191 | hsa-miR-151-3p | hsa-miR-484 |
| hsa-let-7c | hsa-miR-19a | hsa-miR-30a | hsa-miR-106b | hsa-miR-192 | hsa-miR-151-5p | hsa-miR-505 |
| hsa-let-7d | hsa-miR-19b | hsa-miR-30b | hsa-miR-107 | hsa-miR-194 | hsa-miR-186 | hsa-miR-532-5p |
| hsa-let-7e | hsa-miR-20a | hsa-miR-30c | hsa-miR-125a-5p | hsa-miR-196a | hsa-miR-320a | hsa-miR-548b-3p |
| hsa-let-7f | hsa-miR-21 | hsa-miR-30d | hsa-miR-125b | hsa-miR-196b | hsa-miR-324-5p | hsa-miR-590-3p |
| hsa-let-7g | hsa-miR-22 | hsa-miR-30e | hsa-miR-126 | hsa-miR-218 | hsa-miR-331-3p | hsa-miR-598 |
| hsa-let-7i | hsa-miR-23b | hsa-miR-32 | hsa-miR-128 | hsa-miR-221 | hsa-miR-339-5p | hsa-miR-615-3p |
| hsa-miR-7 | hsa-miR-24 | hsa-miR-33a | hsa-miR-130a | hsa-miR-222 | hsa-miR-340 | hsa-miR-652 |
| hsa-miR-9 | hsa-miR-25 | hsa-miR-34a | hsa-miR-130b | hsa-miR-301a | hsa-miR-342-3p | hsa-miR-744 |
| hsa-miR-10a | hsa-miR-26a | hsa-miR-92a | hsa-miR-148a | hsa-miR-301b | hsa-miR-361-5p | hsa-miR-769-5p |
| hsa-miR-10b | hsa-miR-26b | hsa-miR-93 | hsa-miR-148b | hsa-miR-424 | hsa-miR-374a |  |
| hsa-miR-15a | hsa-miR-27a | hsa-miR-96 | hsa-miR-181a | hsa-miR-425 | hsa-miR-374b |  |
| hsa-miR-15b | hsa-miR-27b | hsa-miR-99a | hsa-miR-181c | hsa-miR-454 | hsa-miR-378 |  |
| hsa-miR-16 | hsa-miR-29a | hsa-miR-99b | hsa-miR-182 |  | hsa-miR-421 |  |
