# Supplementary material for: Optimal Use of Conservation and Accessibility Filters in MicroRNA Target Prediction
Source: PLoS One. 2012 Feb 27;7(2):e32208. doi: 10.1371/journal.pone.0032208 (PMC3288066; doi:10.1371/journal.pone.0032208)
Supplement: Table S2 — Overall sensitivity and precision obtained with PACCMIT and other methods. (DOC) [file pone.0032208.s005.doc]

**Table S2.** Overall sensitivity and precision obtained with PACCMIT and other methods.

| **Highly Conserved miRNAs** | | | **Weakly conserved miRNAs** | | |
| --- | --- | --- | --- | --- | --- |
| **Method** | **Sensitivity** | **Precision** | **Method** | **Sensitivity** | **Precision** |
| PACCMIT Access + Cons | 0.428 | 0.761 | PACCMIT Cons | 0.232 | 0.765 |
| PicTar | 0.304 | 0.761 | PACCMIT Access + Cons | 0.134 | 0.750 |
| ElMMo | 0.663 | 0.749 | ElMMo | 0.161 | 0.667 |
| PACCMIT Cons | 0.581 | 0.739 | DIANAmicroT | 0.116 | 0.650 |
| DIANAmicroT | 0.563 | 0.736 | MirSVR | 0.732 | 0.626 |
| MirSVR | 0.897 | 0.620 | TargetScan context score | 0.830 | 0.541 |
| PACCMIT Access | 0.756 | 0.536 | PACCMIT Access | 0.696 | 0.534 |
| Miranda | 0.967 | 0.511 | Miranda | 0.964 | 0.502 |
| PITA | 0.982 | 0.509 | IntaRNA | 0.964 | 0.502 |
| IntaRNA | 0.977 | 0.508 | PITA | 0.964 | 0.500 |
| TargetScan - context score | 0.263 | 0.462 |  |  |  |
| TargetScan - Pct | 0.264 | 0.461 |  |  |  |

The different methods are ranked by precision.
