# Supplementary material for: Optimal Use of Conservation and Accessibility Filters in MicroRNA Target Prediction
Source: PLoS One. 2012 Feb 27;7(2):e32208. doi: 10.1371/journal.pone.0032208 (PMC3288066; doi:10.1371/journal.pone.0032208)
Supplement: Table S3 — Statistical significance of the differences between the four configurations of PACCMIT. P-values were obtained from a one-sided t test and correspond to the null hypothesis. (DOC) [file pone.0032208.s006.doc]

**Table S3.** Statistical significance of the differences between the four configurations of PACCMIT. *P*-values were obtained from a one-sided *t* test and correspond to the null hypothesis.

| **Highly conserved miRNAs** | | | | | | |
| --- | --- | --- | --- | --- | --- | --- |
|  |  |  | **True positives** | | **Precision** | |
| **Predictions per miRNA** | **Method** *A* | **Method** *B* | ***P*-value I***^a^* | ***P*-value II***^b^* | ***P*-value I***^a^* | ***P*-value II***^b^* |
| 2 | No filter | Accessibility | 0.174 | 0.826 | 0.356 | 0.644 |
| 2 | No filter | Conservation | 0.033 | 0.967 | 0.107 | 0.893 |
| 2 | No filter | Access + Cons | 0.009 | 0.991 | 0.062 | 0.938 |
| 2 | Accessibility | Conservation | 0.056 | 0.944 | 0.153 | 0.847 |
| 2 | Accessibility | Access + Cons | 0.014 | 0.986 | 0.079 | 0.921 |
| 2 | Conservation | Access + Cons | 0.492 | 0.508 | 0.187 | 0.813 |
| 4 | No filter | Accessibility | 0.587 | 0.413 | 0.489 | 0.511 |
| 4 | No filter | Conservation | 0.315 | 0.685 | 0.185 | 0.815 |
| 4 | No filter | Access + Cons | 0.033 | 0.967 | 0.101 | 0.899 |
| 4 | Accessibility | Conservation | 0.299 | 0.701 | 0.127 | 0.873 |
| 4 | Accessibility | Access + Cons | 0.052 | 0.948 | 0.056 | 0.944 |
| 4 | Conservation | Access + Cons | 0.078 | 0.922 | 0.069 | 0.931 |
| 8 | No filter | Accessibility | 0.923 | 0.077 | 0.410 | 0.590 |
| 8 | No filter | Conservation | 0.133 | 0.867 | 0.192 | 0.808 |
| 8 | No filter | Access + Cons | 0.030 | 0.970 | 0.059 | 0.941 |
| 8 | Accessibility | Conservation | 0.005 | 0.995 | 0.278 | 0.722 |
| 8 | Accessibility | Access + Cons | 0.008 | 0.992 | 0.086 | 0.914 |
| 8 | Conservation | Access + Cons | 0.049 | 0.951 | 0.005 | 0.995 |
| 10 | No filter | Accessibility | 0.798 | 0.202 | 0.495 | 0.505 |
| 10 | No filter | Conservation | 0.030 | 0.970 | 0.171 | 0.829 |
| 10 | No filter | Access + Cons | 0.006 | 0.994 | 0.069 | 0.931 |
| 10 | Accessibility | Conservation | 0.017 | 0.983 | 0.177 | 0.823 |
| 10 | Accessibility | Access + Cons | 0.004 | 0.996 | 0.072 | 0.928 |
| 10 | Conservation | Access + Cons | 0.102 | 0.898 | 0.052 | 0.948 |
| 20 | No filter | Accessibility | 0.871 | 0.129 | 0.459 | 0.541 |
| 20 | No filter | Conservation | 0.004 | 0.996 | 0.041 | 0.959 |
| 20 | No filter | Access + Cons | 0.004 | 0.996 | 0.041 | 0.959 |
| 20 | Accessibility | Conservation | 0.001 | 0.999 | 0.016 | 0.984 |
| 20 | Accessibility | Access + Cons | < 0.001 | > 0.999 | 0.015 | 0.985 |
| 20 | Conservation | Access + Cons | 0.737 | 0.263 | 0.506 | 0.494 |
| 30 | No filter | Accessibility | 0.986 | 0.014 | 0.610 | 0.390 |
| 30 | No filter | Conservation | 0.002 | 0.998 | 0.020 | 0.980 |
| 30 | No filter | Access + Cons | 0.003 | 0.997 | 0.020 | 0.980 |
| 30 | Accessibility | Conservation | < 0.001 | > 0.999 | 0.001 | 0.999 |
| 30 | Accessibility | Access + Cons | < 0.001 | > 0.999 | < 0.001 | > 0.999 |
| 30 | Conservation | Access + Cons | 0.843 | 0.157 | 0.663 | 0.337 |
| 40 | No filter | Accessibility | 0.992 | 0.008 | 0.911 | 0.089 |
| 40 | No filter | Conservation | < 0.001 | > 0.999 | 0.024 | 0.976 |
| 40 | No filter | Access + Cons | 0.001 | 0.999 | 0.093 | 0.907 |
| 40 | Accessibility | Conservation | < 0.001 | > 0.999 | < 0.001 | > 0.999 |
| 40 | Accessibility | Access + Cons | < 0.001 | > 0.999 | 0.001 | 0.999 |
| 40 | Conservation | Access + Cons | 0.995 | 0.005 | 0.982 | 0.018 |
| Weakly conserved miRNAs | | | | | | |
| 25 | No filter | Accessibility | 0.281 | 0.719 | 0.281 | 0.719 |
| 25 | No filter | Conservation | 0.187 | 0.813 | 0.094 | 0.906 |
| 25 | No filter | Access + Cons | 0.281 | 0.719 | 0.319 | 0.681 |
| 25 | Accessibility | Conservation | 0.500 | 0.500 | 0.259 | 0.741 |
| 25 | Accessibility | Access + Cons | 0.500 | 0.500 | 0.574 | 0.426 |
| 25 | Conservation | Access + Cons | 0.500 | 0.500 | 0.813 | 0.187 |
| 50 | No filter | Accessibility | 0.110 | 0.890 | 0.112 | 0.888 |
| 50 | No filter | Conservation | 0.517 | 0.483 | 0.402 | 0.598 |
| 50 | No filter | Access + Cons | 0.512 | 0.488 | 0.842 | 0.158 |
| 50 | Accessibility | Conservation | 0.857 | 0.143 | 0.678 | 0.322 |
| 50 | Accessibility | Access + Cons | 0.816 | 0.184 | 0.941 | 0.059 |
| 50 | Conservation | Access + Cons | 0.500 | 0.500 | 0.806 | 0.194 |
| 75 | No filter | Accessibility | 0.086 | 0.914 | 0.167 | 0.833 |
| 75 | No filter | Conservation | 0.773 | 0.227 | 0.642 | 0.358 |
| 75 | No filter | Access + Cons | 0.773 | 0.227 | 0.677 | 0.323 |
| 75 | Accessibility | Conservation | 0.943 | 0.057 | 0.781 | 0.219 |
| 75 | Accessibility | Access + Cons | 0.943 | 0.057 | 0.808 | 0.192 |
| 75 | Conservation | Access + Cons | 0.500 | 0.500 | 0.527 | 0.473 |
| 100 | No filter | Accessibility | 0.056 | 0.944 | 0.105 | 0.895 |
| 100 | No filter | Conservation | 0.794 | 0.206 | 0.653 | 0.347 |
| 100 | No filter | Access + Cons | 0.864 | 0.136 | 0.522 | 0.478 |
| 100 | Accessibility | Conservation | 0.921 | 0.079 | 0.767 | 0.233 |
| 100 | Accessibility | Access + Cons | 0.960 | 0.040 | 0.773 | 0.227 |
| 100 | Conservation | Access + Cons | 0.450 | 0.550 | 0.368 | 0.632 |

*^a^* Alternative hypothesis: true difference in means (Method *B* – Method *A*) is greater than 0.

*^b^* Alternative hypothesis: true difference in means (Method *A* – Method *B*) is greater than 0.
