# Supplementary material for: Optimal Use of Conservation and Accessibility Filters in MicroRNA Target Prediction
Source: PLoS One. 2012 Feb 27;7(2):e32208. doi: 10.1371/journal.pone.0032208 (PMC3288066; doi:10.1371/journal.pone.0032208)
Supplement: Table S4 — Statistical significance of the differences between PACCMIT and other methods. P-values were obtained from a one-sided t test and correspond to the null hypothesis. (DOC) [file pone.0032208.s007.doc]

**Table S4.** Statistical significance of the differences between PACCMIT and other methods. *P*-values were obtained from a one-sided *t* test and correspond to the null hypothesis.

| **Hightly conserved miRNAs** | | | | |
| --- | --- | --- | --- | --- |
|  | | | **True positives** | **Precision** |
| **Predictions per miRNA** | **Method** *A* | **Method** *B* | ***P*-value***^a^* | ***P*-value***^a^* |
| 2 | PACCMIT Access + Cons | TargetScan-score | 0.071 | 0.058 |
| 2 | PACCMIT Access + Cons | TargetScan-Pct | 0.156 | 0.187 |
| 2 | PACCMIT Access + Cons | DIANA-microT | 0.956 | 0.187 |
| 2 | PACCMIT Access + Cons | MirSVR | 0.000 | N.D |
| 2 | PACCMIT Access + Cons | PicTar | 0.127 | 0.071 |
| 2 | PACCMIT Access + Cons | ElMMo | <0.001 | 0.058 |
| 2 | PACCMIT Access + Cons | Miranda | 0.001 | 0.187 |
| 2 | PACCMIT Access + Cons | PITA | <0.001 | 0.058 |
| 2 | PACCMIT Access + Cons | IntaRNA | <0.001 | N.D |
| 4 | PACCMIT Access + Cons | TargetScan-score | 0.207 | 0.006 |
| 4 | PACCMIT Access + Cons | TargetScan-Pct | 0.117 | 0.278 |
| 4 | PACCMIT Access + Cons | DIANA-microT | 0.967 | 0.052 |
| 4 | PACCMIT Access + Cons | MirSVR | <0.001 | <0.001 |
| 4 | PACCMIT Access + Cons | PicTar | 0.134 | 0.137 |
| 4 | PACCMIT Access + Cons | ElMMo | 0.003 | 0.001 |
| 4 | PACCMIT Access + Cons | Miranda | 0.009 | 0.029 |
| 4 | PACCMIT Access + Cons | PITA | 0.001 | 0.941 |
| 4 | PACCMIT Access + Cons | IntaRNA | <0.001 | <0.001 |
| 8 | PACCMIT Access + Cons | TargetScan-score | 0.183 | 0.061 |
| 8 | PACCMIT Access + Cons | TargetScan-Pct | 0.027 | 0.067 |
| 8 | PACCMIT Access + Cons | DIANA-microT | 0.780 | 0.377 |
| 8 | PACCMIT Access + Cons | MirSVR | 0.004 | 0.018 |
| 8 | PACCMIT Access + Cons | PicTar | 0.186 | 0.294 |
| 8 | PACCMIT Access + Cons | ElMMo | 0.004 | 0.008 |
| 8 | PACCMIT Access + Cons | Miranda | 0.009 | 0.006 |
| 8 | PACCMIT Access + Cons | PITA | 0.001 | 0.167 |
| 8 | PACCMIT Access + Cons | IntaRNA | 0.001 | 0.006 |
| 10 | PACCMIT Cons | TargetScan-score | 0.743 | 0.443 |
| 10 | PACCMIT Cons | TargetScan-Pct | 0.120 | 0.703 |
| 10 | PACCMIT Cons | DIANA-microT | 0.921 | 0.960 |
| 10 | PACCMIT Cons | MirSVR | 0.014 | 0.018 |
| 10 | PACCMIT Cons | PicTar | 0.405 | 0.593 |
| 10 | PACCMIT Cons | ElMMo | 0.004 | 0.156 |
| 10 | PACCMIT Cons | Miranda | 0.013 | 0.140 |
| 10 | PACCMIT Cons | PITA | <0.001 | 0.043 |
| 10 | PACCMIT Cons | IntaRNA | <0.001 | 0.197 |
| 20 | PACCMIT Cons | TargetScan-score | 0.087 | 0.612 |
| 20 | PACCMIT Cons | TargetScan-Pct | 0.029 | 0.221 |
| 20 | PACCMIT Cons | DIANA-microT | 0.542 | 0.936 |
| 20 | PACCMIT Cons | MirSVR | 0.004 | 0.008 |
| 20 | PACCMIT Cons | PicTar | 0.206 | 0.754 |
| 20 | PACCMIT Cons | ElMMo | <0.001 | 0.089 |
| 20 | PACCMIT Cons | Miranda | 0.002 | 0.416 |
| 20 | PACCMIT Cons | PITA | <0.001 | 0.083 |
| 20 | PACCMIT Cons | IntaRNA | <0.001 | 0.007 |
| 40 | PACCMIT Cons | TargetScan-score | 0.008 | 0.548 |
| 40 | PACCMIT Cons | TargetScan-Pct | <0.001 | 0.872 |
| 40 | PACCMIT Cons | DIANA-microT | 0.201 | 0.897 |
| 40 | PACCMIT Cons | MirSVR | 0.003 | 0.032 |
| 40 | PACCMIT Cons | PicTar | 0.147 | 0.887 |
| 40 | PACCMIT Cons | ElMMo | <0.001 | 0.085 |
| 40 | PACCMIT Cons | Miranda | <0.001 | 0.147 |
| 40 | PACCMIT Cons | PITA | <0.001 | 0.009 |
| 40 | PACCMIT Cons | IntaRNA | <0.001 | 0.000 |
| **Weakly conserved miRNAs** | | | | |
| 25 | PACCMIT Access | TargetScan-score | 0.678 | 0.678 |
| 25 | PACCMIT Access | DIANA-microT | 0.187 | N.D |
| 25 | PACCMIT Access | MirSVR | 0.399 | 0.638 |
| 25 | PACCMIT Access | ElMMo | 0.187 | N.D |
| 25 | PACCMIT Access | Miranda | 0.187 | N.D |
| 25 | PACCMIT Access | PITA | 0.187 | N.D |
| 25 | PACCMIT Access | IntaRNA | 0.398 | 0.500 |
| 50 | PACCMIT Access | TargetScan-score | 0.733 | 0.881 |
| 50 | PACCMIT Access | DIANA-microT | 0.203 | 0.921 |
| 50 | PACCMIT Access | MirSVR | 0.100 | 0.292 |
| 50 | PACCMIT Access | ElMMo | 0.027 | 0.003 |
| 50 | PACCMIT Access | Miranda | 0.092 | 0.109 |
| 50 | PACCMIT Access | PITA | 0.041 | 0.158 |
| 50 | PACCMIT Access | IntaRNA | 0.088 | 0.415 |
| 100 | PACCMIT Access | TargetScan-score | 0.505 | 0.350 |
| 100 | PACCMIT Access | DIANA-microT | 0.011 | 0.468 |
| 100 | PACCMIT Access | MirSVR | 0.025 | 0.109 |
| 100 | PACCMIT Access | ElMMo | 0.005 | 0.100 |
| 100 | PACCMIT Access | Miranda | 0.009 | 0.080 |
| 100 | PACCMIT Access | PITA | 0.002 | 0.100 |
| 100 | PACCMIT Access | IntaRNA | 0.011 | 0.309 |

*^a^* Alternative hypothesis: true difference in means (Method *A* – Method *B*) is greater than 0.

*^b^* Not defined.
